# Rapid One-Pot Synthesis of Polydopamine Encapsulated Carbon Anchored with Au Nanoparticles: Versatile Electrocatalysts for Chloramphenicol and Folic Acid Sensors

**DOI:** 10.3390/ijms21082853

**Published:** 2020-04-19

**Authors:** Veerappan Mani, T.S.T. Balamurugan, Sheng-Tung Huang

**Affiliations:** Institute of Biochemical and Biomedical Engineering, Department of Chemical Engineering and Biotechnology, National Taipei University of Technology, No.1, Section 3, Chung-Hsiao East Road, Taipei 10608, Taiwan

**Keywords:** biopolymer, green synthesis, electroanalysis, electrocatalytic sensor, vitamins, antibiotics

## Abstract

Designing and engineering nanocomposites with tailored physiochemical properties through teaming distinct components is a straightforward strategy to yield multifunctional materials. Here, we describe a rapid, economical, and green one-pot microwave synthetic procedure for the preparation of ternary nanocomposites carbon/polydopamine/Au nanoparticles (C/PDA/AuNPs; C = carbon nanotubes (CNTs), reduced graphene oxide (rGO)). No harsh reaction conditions were used in the method, as are used in conventional hydrothermal or high-temperature methods. The PDA unit acts as a non-covalent functionalizing agent for carbon, through π stacking interactions, and also as a stabilizing agent for the formation of AuNPs. The CNTs/PDA/AuNPs modified electrode exhibited excellent electrocatalytic activity to oxidize chloramphenicol and the resulting sensor exhibited a low detection limit (36 nM), wide linear range (0.1–534 μM), good selectivity (against 5-fold excess levels of interferences), appreciable reproducibility (3.47%), good stability (94.7%), and practicality (recoveries 95.0%–98.4%). Likewise, rGO/PDA/AuNPs was used to fabricate a sensitive folic acid sensor, which exhibits excellent analytical parameters, including wide linear range (0.1–905 μM) and low detection limit (25 nM). The described synthetic route includes fast reaction time (5 min) and a readily available household microwave heating device, which has the potential to significantly contribute to the current state of the field.

## 1. Introduction

Nano architectures hold a significant role in modern technology, with a wide range of applications in clinical diagnosis, drug delivery, catalysis, energy storage, and electroanalytical science [1,2,3]. This wide array of utilities is made possible by tailoring the physiochemical properties of nanostructures, which can be done by coupling two or more nanostructures to engineer a fruitful nanocomposite with the desired physiochemical properties [4,5]. Designing and engineering nanocomposites with tailor-made physiochemical properties through teaming distinct nanocomponents results in enhanced mechanical strength, abundant surface area, high charge storage capacity, improved electrical conductivity, improved stability, biocompatibility, and elevated electrocatalytic performance [6,7,8]. Carbon nanostructures have become well known over the years thanks to their unique structures, good chemical stability, high aspect ratio, low density, and excellent electronic, thermal, and mechanical behaviors [9]. Despite these unique physiochemical attributes, pristine carbon nanostructures like graphene and carbon nanotubes (CNTs) are not easy to access in a wide range of applications [10]. Thus, tailoring the carbon materials via functionalization has become essential in order to refine their electronic properties for a desired application [6,11]. Interestingly, carbon nanomaterials are easy to be functionalized and act as a fruitful supporting backbone for metals and metal oxides [12,13]. This property enables a range of possibilities to design carbon nanomaterials functionalized and/or anchored with various nanostructures aimed at specific applications [14]. Carbon materials bound with polymers and/or metal nanoparticles were applied distinctly for various applications like batteries, supercapacitors, and electrocatalysis [15]. The polymer functionalization of carbon materials has derived considerable attention due to the fact of their uniform encapsulation/functionalization over the surface of carbon backbone and offers additional active sites for catalysis and secondary functionalization [15]. Added to this, calcination of heterotopic polymer-functionalized carbon materials offers a unique entrapment of heterotopic graphene sheet over primary carbon backbone with abundant active sites and improved catalytic attributes [16]. Polydopamine (PDA) is a key bio-mimic polymer employed to functionalize carbon materials, courtesy of its excellent biocompatibility, uniform entrapment on the surface, controllable polymerization thickness, abundant functional groups for catalysis, and secondary functionalization options [15]. PDA coating can be easily prepared by self-polymerization of dopamine at alkaline pH, and this coating can be used to immobilize a wide variety of molecules, such as active metal nanoparticles [17]. The polymerization is a quick process and the coating thickness can be controlled by polymerization time. Metal nanoparticles have become widely known over the years and are employed in a broad range of utilities in electroanalysis [18,19]. The inherent physicochemical attributes along with the excellent synergy on blending with carbon nanostructures with customized physiochemical assets for specific functions has elevated their real time utilities vividly over the years [20]. The idea of ternary carbon nanocomposites derived from primarily functionalized carbon nanostructures are an evolutionary sequence to design carbon nanomaterials with customized physiochemical attributes for a definite function [13,21]. The polymer enwrapped carbon nanostructures anchored with metal nanoparticles is an effective way to tailor the physiochemical attributes of the nanostructures for specific applications, as the composites have the synergic blend of the all three constituents [19]. The design of carbon enwrapped PDA bounded with metal nanostructures (C/PDA/MNP) are of recent interest due to its wide array of applications in diverse areas of analytical chemistry. For instance, Li et al. prepared CNTs/PDA/gold nanoparticles (AuNPs) as an electrocatalyst for simultaneous detection of catechol and hydroquinone [22]. Liu et al. designed Ag–PDA/graphene as an electrocatalyst to assay adenine and guanine [23]. Wang et al. constructed a DNA biosensor using AgNPs-PDA/graphene as an electrocatalyst [24]. Liang et al. reported a hydrogen peroxide sensor based on graphene oxide (GO)/PDA/Cu NPs electrode [25]. Yao et al. employed AuNPs-PDA-reduced GO catalyst to build an immunosensor to quantify immunoglobulin G [26]. Despite their strong electrocatalytic proficiency, the design and preparations of such ternary nanocomposites are quite troublesome as it involves multiples steps, harsh reagents, use of high pressure or temperature, and prolonged reaction time [24].

**Scheme 1:**
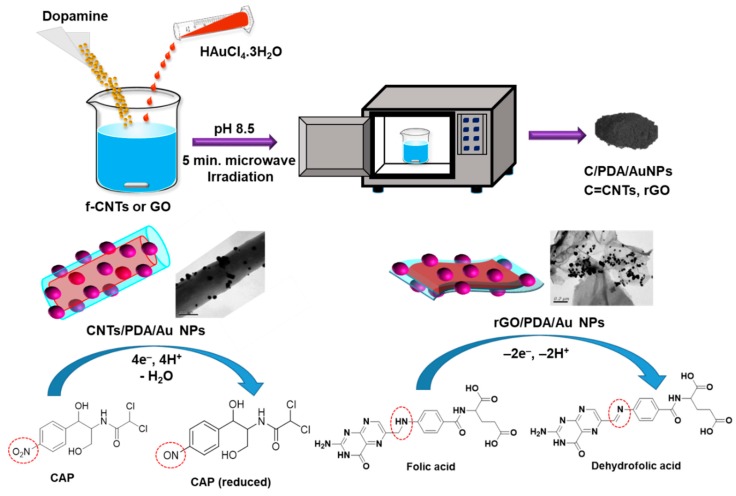
Schematic representation for rapid synthesis of C/PDA/AuNPs; C = CNTs & rGO, PDA = Polydopamine, AuNPs = Gold nanoparticles (top). (bottom) Structural architecture and TEM images of the composites.

In this study, we are proposing an alternate microwave-assisted green synthetic protocol to prepare PDA-encapsulated carbon nanomaterials anchored with AuNPs. The synthesis involves a rapid one-step polymerization of PDA over carbon nanomaterials followed by a growth of AuNPs over PDA enwrapped carbon backbone (Scheme 1). Efficacy of the synthetic route has been demonstrated via preparing a pair of electrocatalysts, (1) CNTs/PDA/AuNPs and (2) rGO/PDA/AuNPs; Here PDA = Polydopamine, rGO = reduced graphene oxide. Both the nanocomposites have excellent surface properties, porous structures, high conductivity, large electrochemical surface area, and excellent electrocatalytic abilities. The main advantages of the method are fast reaction time and the use of a readily available household microwave heating device. The physicochemical properties of the CNTs/PDA/AuNPs and rGO/PDA/AuNPs nanocomposites have been used to fabricate electrochemical sensors for chloramphenicol (CAP) and folic acid (FA), respectively. Chloramphenicol is a veterinary antibacterial drug commonly used to treat infectious diseases in food-producing animals [27,28]. However, its excess level is associated with serious toxic effects such as bone marrow depression and its absorption in human beings is rapid and extensive after an oral dose [29,30,31]. Hence, its quantitative determination in foods that originate from poultry is essential. Electrochemical sensors are robust, inexpensive, portable, and easy to operate [32]. Here, we have developed a sensitive CAP sensor using the CNTs/PDA/AuNPs nanocomposite-modified electrode. Folic acid (FA) is a B-group vitamin that plays a significant role in the biological functions of cell metabolism [33,34]. Its deficiency in our body causes many disorders, including increased risk of colorectal cancer, neural tube defects, hypomethylation, and triggering proto-oncogene expression in cancer [35,36,37]. Supplements in different quantities of FA are available to treat FA deficiency and hence its quantitative determination is necessary to control the quality of pharmaceutical and food products. Using the rGO/PDA/AuNPs nanocomposite, here, we have developed a handy and affordable electrochemical sensor for detecting FA in food samples.

## 2. Results and Discussion

### 2.1. The Morphology, Structure, and Composition of CNTs/PDA/AuNPs

**Figure 1:**
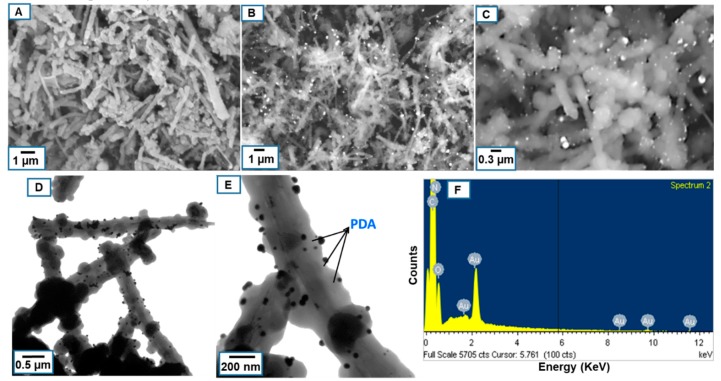
(**A**) SEM images of CNTs/PDA and CNTs/PDA/AuNPs at (**B**) lower and (**C**) higher magnifications. (**D**,**E**) TEM image of CNTs/PDA/AuNPs at different magnifications. (**F**) EDX spectrum of CNTs/PDA/AuNPs.

The morphology of the materials was assessed by SEM and TEM analyses. The SEM micrograph of CNTs/PDA displays the characteristic tubular morphology of CNTs; however, the PDA coating is not clearly visible (Figure 1A). The image of CNTs/PDA/AuNPs reveals the attachment of a large number of spherical particles in the hierarchal interconnected networks of CNTs/PDA (Figure 1B). The formation of nanoparticles is apparent from the higher magnification SEM as the particle sizes are in the nanometer scale (Figure 1C). The morphology of the composite was further viewed by TEM at different magnifications, which clearly revealed the distribution of minute nanosized spherical AuNPs on the surface of CNTs/PDA (Figure 1D,E). No particle is dispersed outside the matrix of CNTs/PDA. In the TEM image, the coatings of PDA appear as non-smooth coatings on the walls of CNTs. The appearance of AuNPs attachment indicates that the growth of each particle has originated from the PDA layer, thus PDA is an appropriate stabilizing agent for AuNPs. The EDX profile of CNTs/PDA/AuNPs reveals the signals of expected elements, C, N, O, and Au (Figure 1F), with atomic percentages of 79.54%, 9.42%, 10.5%, and 0.54%, and weight percentages of 71.14%, 9.83%, 12.63%, and 6.4%, respectively.

**Figure 2:**
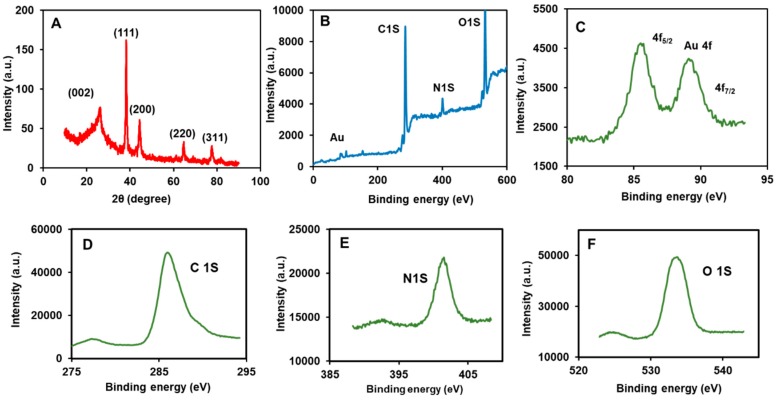
(**A**) X-ray diffraction patterns, (**B**) X-ray photoelectron spectroscopy (XPS) survey spectrum, and (**C**–**F**) XPS relevant to each atom of the CNTs/PDA/AuNPs nanocomposite.

Figure 2A displays the powder XRD patterns of the CNTs/PDA/AuNPs. The diffraction peak that appeared at the 2θ angle of 26.2 can be assigned to the hexagonal crystalline graphite. The diffraction peaks observed at 38.7, 44.5, 64.8, and 77.8 can be allocated to Au (111), (200), (220), and (311) respectively, which confirms the presence of AuNPs in the composite [38,39]. Next, the surface chemical compositions were investigated using XPS (Figure 2B). The XPS spectrum of CNTs/PDA/AuNPs displayed the characteristic XPS signals at the binding energies of 85.6, 89.2, 286.2, 401.4, and 533.7 eV. The peaks appearing at 85.6 and 89.2 eV were corresponding to the 4f5_/2_ and 4f7_/2_ spin-orbit split components, characteristic of the presence of the Au element in the composite (Figure 2C). The XPS signal originating at 286.2 eV can be associated with the C 1s mode, which originates from the graphitic network of reduced graphene oxide (Figure 2D). The XPS peak which appeared at 401.4 matches with the XPS signature of N1s (Figure 2E). The signal observed at 532.5 eV can be correlated to the O 1s that originated from the PDA and to the residual oxygen functionalities incorporated in the rGO sheets (Figure 2F).

### 2.2. Characterizations of rGO/PDA/AuNPs

**Figure 3:**
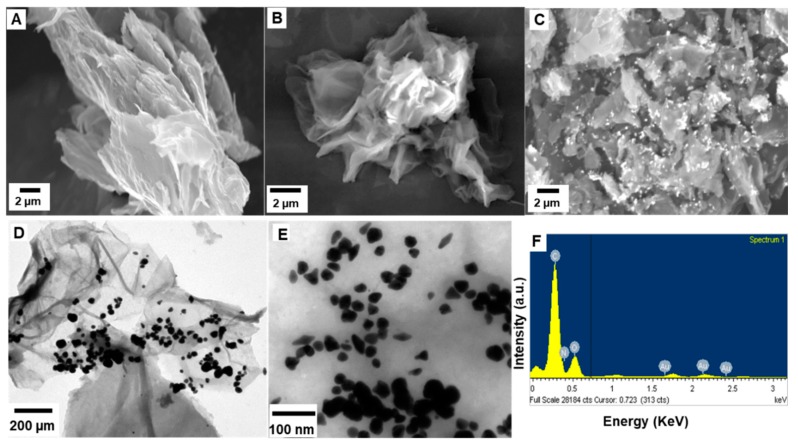
(**A**) SEM images of rGO, (**B**) rGO/PDA, and (**C**) rGO/PDA/AuNPs. (**D**,**E**) TEM images of rGO/PDA/AuNPs at different magnifications. (**F**) EDX spectrum of rGO/PDA/AuNPs.

Next, the formation of the rGO/PDA/AuNPs nanocomposite was verified by characterization studies. The SEM micrograph of rGO portrays a characteristic wrinkled sheets-like morphology of rGO (Figure 3A). The coating of PDA in layers of rGO sheets is not clear in the SEM of rGO/PDA, which could be due to the poor conducting property of PDA (Figure 3B). However, the presence of abundant AuNPs onto the sheets is evident in the SEM of rGO/PDA/AuNPs (Figure 3C). The TEM images of rGO/PDA/AuNPs at different magnifications revealed the distribution of the nanosized Au particles on the rGO/PDA sheets (Figure 3D,E). The EDX profile of rGO/PDA/AuNPs shows the elements C, O, N, and Au (Figure 3F), with atomic percentages of 69.70%, 23.33%, 6.35%, and 0.62%, and weight percentages of 66.81%, 24.29%, 6.24%, and 2.66%, respectively.

**Figure 4:**
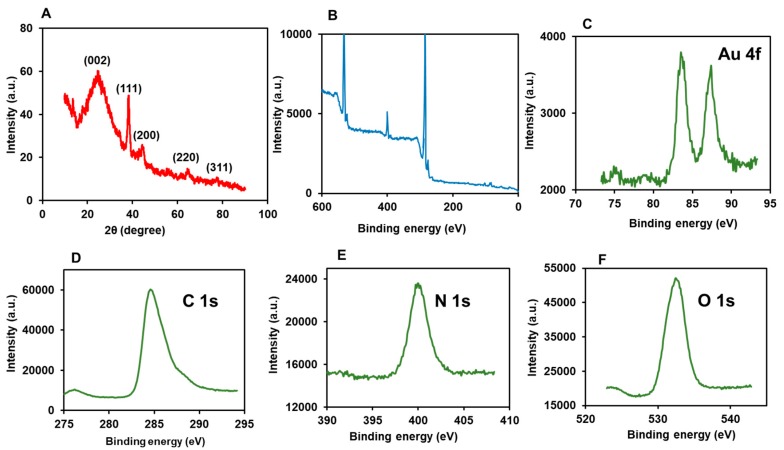
(**A**) XRD, (**B**) XPS survey, and (**C**–**F**) XPS relevant to atoms of the rGO/PDA/AuNPs composite.

Figure 4A shows the XRD profile of the rGO/PDA/AuNPs. The diffraction peak at 25.1 is assigned to the graphite structure of rGO, and the signals at 38.4 (111), 44.1(200), 64.7 (220), and 77.2 (311) correspond to the pattern of AuNPs [40]. The XPS spectrum of rGO/PDA/AuNPs displayed the characteristic signals at 83.2, 87.3, 284.8, 400.2, and 532.6 eV (Figure 4B). The 4f5_/2_ and 4f7_/2_ spin-orbit split components at 83.2 and 87.3 eV respectively, indicate the presence of Au (Figure 4C). A signal at 284.8 eV is identified as a C 1s mode, originating from the graphitic network of the rGO (Figure 4D). A signal at 400.2 eV relates N1s (Figure 4E) and a signal at 532.6 eV relates O 1s, which originates from PDA (Figure 4F).

### 2.3. Electrocatalytic Sensing Ability of the CNTs/PDA/AuNPs-Modified Electrode to Chloramphenicol

**Figure 5:**
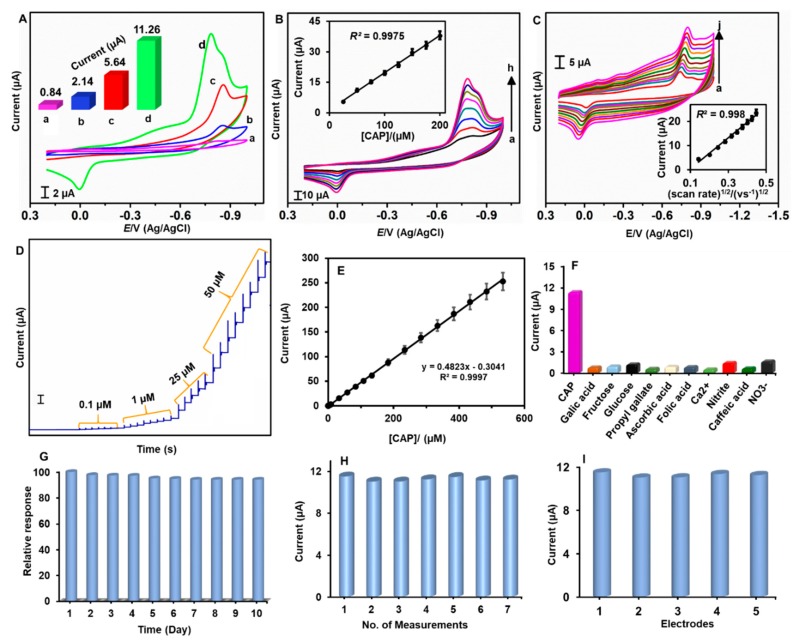
(**A**) Cyclic voltammograms (CVs) at a scan rate of 0.05 V s^−1^ obtained for (a) unmodified, (b) CNTs/PDA, (c) CNTs/AuNPs, and (d) CNTs/PDA/AuNPs films-modified glassy carbon electrodes (GCEs) in phosphate buffer (pH 7.0) containing 50 µM of CAP. (**B**) CVs of GCE/CNTs/PDA/AuNPs in phosphate buffer (pH 7.0) with varied concentrations of CAP (a = 25, b = 50, c = 75, d = 100, e = 125, f = 150, g = 175, and h = 200 µM). Inset: cathodic peak current/µA versus [CAP]/µM. (**C**) CVs at different scan rates (a to j: 0.02 to 0.2 V s^−1^) of GCE/CNTs/PDA/AuNPs in phosphate buffer (pH 7.0) containing 50 µM CAP. Inset: CAP reduction peak current versus (scan rate)^1/2^. (**D**) Amperometric response of GCE/CNTs/PDA/AuNPs to varied amounts of CAP, injected in phosphate buffer (pH 7.0), and (**E**) corresponding linear calibration plot. (**F**) Amperometric responses of GCE/CNTs/PDA/AuNPs to 50 µM CAP and 0.1 mM of other analytes. (**G**) CAP sensing performance of GCE/CNTs/PDA/AuNPs for 10 continuous days (stability test), (**H**) 7 repeated measurements using a single electrode (repeatability test) and (**I**) reproducibility of 5 separate experiments using 5 separately fabricated electrodes. For stability, repeatability, and reproducibility tests, CVs were performed with 50 µM CAP suspended in 0.1 M phosphate buffer (pH 7.0), while CAP reduction peak current was used to plot the bar diagram.

The electrocatalytic activities of GCE/CNTs/PDA/AuNPs and its control electrodes have been studied by voltammetry (Figure 5A). Here, GCE = glassy carbon electrode. The cathodic peak current (*I*_pc_) responsible for CAP reduction is found to be 13.4, 5.3, and 2 times higher than that obtained at unmodified, GCE/PDA/CNTs, and GCE/CNTs/AuNPs, respectively (inset to Figure 5A). The main cathodic peak is corresponding to the electrocatalytic reduction of the NO_2_ group of CAP to NHOH (hydroxyl) [41]. The NHOH can be further oxidized to NO at positive potentials. At GCE/CNTs/PDA/AuNPs, the overpotential for the CAP reduction reaction is observed 100 mV lower than the unmodified GCE. Improved peak current and decreased overpotential corroborate the superior electrocatalytic aptitude of CNTs/PDA/AuNPs for CAP reduction. Figure 5B presents the voltammetric responses of GCE/CNTs/PDA/AuNPs toward different concentrations of CAP. As the concentration of CAP increased, the *I*_pc_ increased linearly, indicating excellent electrocatalysis (inset to Figure 5B). The good linearity also suggests that the electrode does not suffer from fouling. Figure 5C shows the CV responses of CNTs/PDA/AuNPs toward 50 μM CAP at different scan rates, between 20 and 200 mVs^−1^. The plot between *I*_pc_ and the square root of scan rate exhibited good linearity, indicating diffusion-controlled reduction process of CAP (inset to Figure 5C). Figure 5D presents the amperometric signals of the CNTs/PDA/AuNPs-modified electrode upon successive injections of CAP into phosphate buffer (pH 7.0) at regular intervals of 50 s (*E*_app_ = −0.70 V). The electrode area is 0.21 cm^2^ and the rotation speed is 1200 rpm. Well-defined and quick responses were obtained immediately after injection of CAP. A steady-state current was reached in five seconds, indicating a rapid response time. A linear increase in the current response was observed as shown in the calibration plot. The corresponding linear regression equation is stated as follows: *I*_pc_ (µA) = 1.5737 C (μM) + 26.398. The linear range was 0.1–534 µM and sensitivity was 2.296 μA µM^−1^ cm^−2^ (Figure 5E). The detection limit (LOD) was 36 nM. The sensor parameters are either superior or comparable to the existing modifiers for CAP analysis, as given in Table 1.

Next, selectivity of the modified electrode was evaluated by monitoring its sensing ability to likely interfering agents. Figure 5F displays the amperometric response of CNTs/PDA/AuNPs to 50 μM CAP and 250 μM interfering compounds (food additives, biological analytes, and metals). The amperometric experiments were conducted by successive injections of 50 μM CAP, 250 μM of each interfering compounds, and finally, again with 50 μM CAP at regular intervals of 50 s. The electrode potential was –0.70 V and the electrode rotation speed was 1200 rpm. The electrode responded rapidly to CAP; however, it was insensitive to other analytes such as, gallic acid, fructose, glucose, propyl gallate, ascorbic acid, folic acid, Ca^2+^, and caffeic acid. These results suggest that CNTs/PDA/AuNPs have good specificity for recognizing CAP in the presence of other analytes. Only nitrite and nitrate showed slight interferences; but, their contribution is still less than 5%. The π-stacking interaction between phenyl moieties of the CNTs/PDA and the CAP may play an important role in dictating the selectivity. Other nitrite compounds that do not own π electrons are unable to produce measurable signals. The common biological interfering species such as ascorbic acid and glucose did not show significant interference, because the working potential is away from their oxidization potential region. Next, the durability, repeatability, and reproducibility of the sensor were tested. To assess storage stability, the CV performance of GCE/CNTs/PDA/AuNPs towards CAP (50 µM) was monitored every day (Figure 5G). After 10 days of continuous storage, the electrode retained about 94.7% of its initial response, indicating its good durability. The sensor presented acceptable repeatability with a Relative Standard Deviation (RSD) of 2.85% for seven subsequent measurements carried out using a single CNTs/PDA/AuNPs-modified electrode (Figure 5H). Moreover, the modified electrode retained good reproducibility, as the RSD of five separate measurements on all five electrodes was 3.47% (Figure 5I). Next, practicality of the sensor was demonstrated in food samples. Milk, powdered milk, and honey samples were prepared by following the procedures given in the experimental section. Amperometry experiments were carried out using GCE/CNTs/PDA/AuNPs in CAP-spiked food samples. The spiked CAP concentrations were 1, 5, and 10 µM. The added, found, and recovery values are presented in Table 2. For the three food samples tested, the recovery values were 95.0%–98.4%. Because the recoveries are in an acceptable range, we concluded that the developed modified electrode has good practical applicability and could be a potential sensor in food safety testing.

### 2.4. rGO/PDA/AuNPs Modified Electrode: An Excellent Electrocatalyst for Sensing Folic Acid

**Figure 6:**
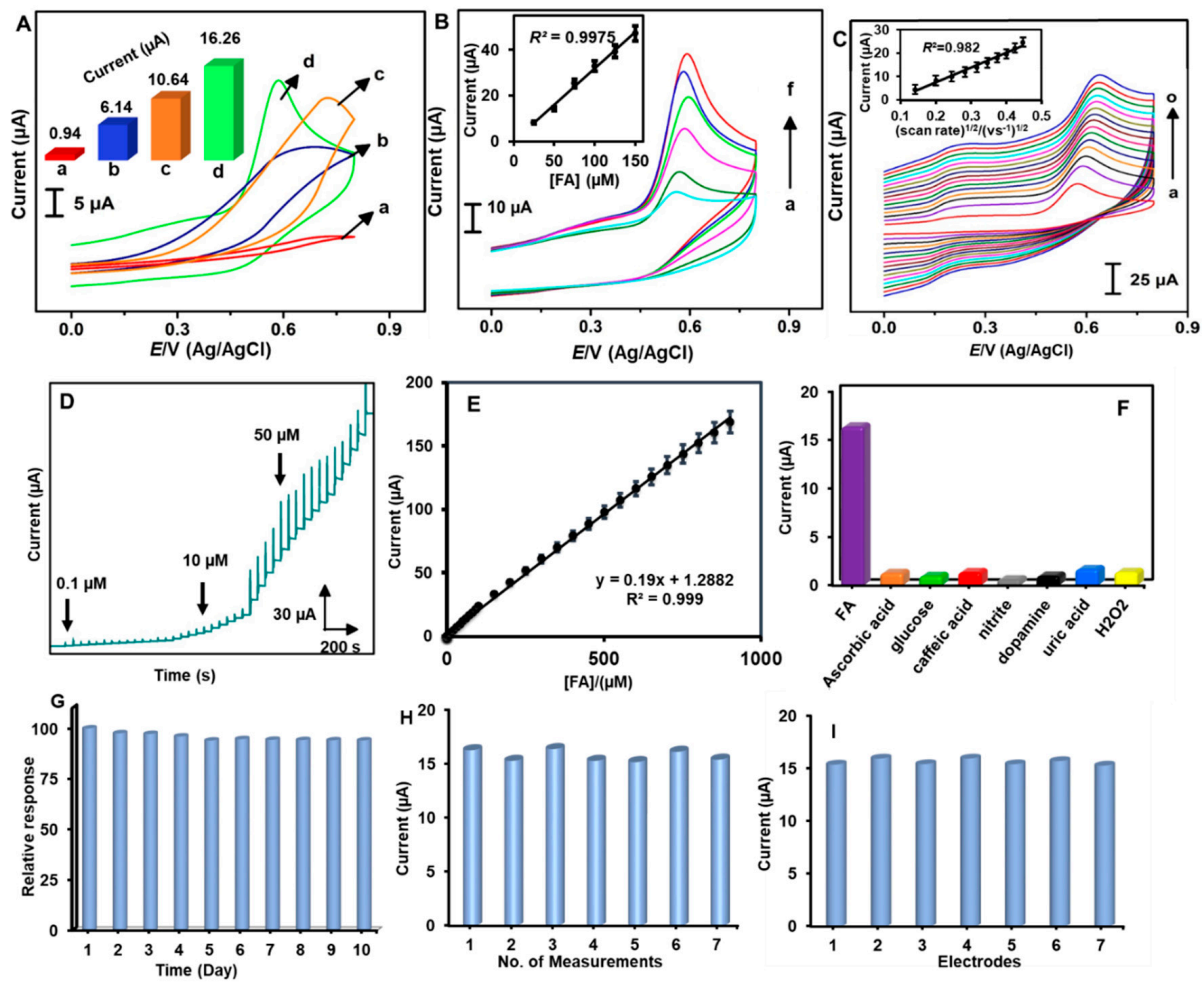
(**A**) CVs obtained at (a) unmodified GCE, (b) GCE/rGO/DA, (c) GCE/rGO/AuNPs, and (d) GCE/rGO/PDA/AuNPs towards 50 µM FA suspended in phosphate buffer (pH 7.0). Inset: plot of current versus electrodes. (**B**) CVs of GCE/rGO/PDA/AuNPs towards varied concentrations of FA (a = 25, b = 50, c = 75, d = 100, e = 125, f = 150 µM) suspended in phosphate buffer (pH 7.0), inset: anodic peak current/µA versus [FA]/µM. (**C**) CVs obtained at GCE/rGO/PDA/AuNPs in phosphate buffer (pH 7.0) containing 50 µM FA at different scan rates (a to j: 0.02 to 0.2 V s^−1^). Inset: FA reduction peak current versus (scan rate)^1/2^. (**D**) Amperometric responses of GCE/rGO/PDA/AuNPs towards series of different FA concentrations injected in phosphate buffer (pH 7.0) and (**E**) corresponding linear calibration plot, applied potential = 0.55 V. (**F**) Amperometric responses of GCE/rGO/PDA/AuNPs toward 50 µM FA and 10-fold higher concentration of other species. (**G**) FA sensing performance of GCE/rGO/PDA/AuNPs for 10 continuous days (stability test), (**H**) 7 repeated measurements using a single electrode (repeatability test) and (**I**) reproducibility of 7 separate experiments using 7 independently fabricated electrodes. For stability, repeatability, and reproducibility, CVs were performed toward 50 µM FA in a 0.1 M phosphate buffer (pH 7.0), while FA oxidation peak current was used to plot the bar diagram.

Next, the electrocatalytic sensing ability of the rGO/PDA/AuNPs composite has been tested for folic acid (FA). Figure 6A presents the CVs of rGO/PDA/AuNPs and its controls toward 50 μM FA. The oxidation peak current (*I*_pa_) of FA at the rGO/PDA/AuNPs-modified GCE was 17.3-, 2.65-, and 1.53-folds higher than those obtained at the unmodified, rGO/PDA, and rGO/AuNPs films-modified electrodes (inset to figure 6A). In addition, compared to unmodified GCE, GCE/rGO/PDA, and GCE/rGO/AuNPs, the rGO/PDA/AuNPs-modified electrode has less overpotential of about 180, 50, and 150 mV, respectively. Thus, the improved peak current and lowered overpotential indicate that the rGO/PDA/AuNPs is a good electrocatalyst to oxidize FA. Figure 6B shows the linear responses of GCE/rGO/PDA/AuNPs to increasing concentrations of FA. Figure 6C presents the voltammetric responses of 50 μM FA at different scan rates (0.02–0.2 Vs^−1^). The plot between the anodic peak current and the square root of scan rate exhibited good linearity, suggesting the diffusion-controlled reduction process of FA. Figure 6D presents the amperometric responses of the rGO/PDA/AuNPs-modified electrode to increasing concentrations of FA. The applied potential was +0.55 V. Quick responses were obtained within 5 s. A wide linear range, 0.1–905 µM, was observed with a linear regression equation of, *I*_pc_ (µA) = 0.19 (FA) (μM) + 1.29, *R*^2^ = 0.999 (Figure 6E). The sensitivity was 2.75 μA µM^−1^ cm^−2^ and the detection limit was 25 nM. The sensor parameters of this electrode were significantly improved compared to existing modified electrodes (Table 3). As shown in Figure 6F, the rGO/PDA/AuNPs is highly selective to FA in the presence of other electroactive analytes. The electrode has good storage stability, as evidenced by the fact that 95.2% of its initial response is retained after 10 days of its use (Figure 6G). Furthermore, the sensor showed acceptable repeatability with an RSD of 3.15% for seven repetitive measurements (Figure 6H), and an appreciable reproducibility with RSD of 3.37% for seven individual electrodes (Figure 6I).

Practicality of the FA sensor was demonstrated in human serum and urine samples. First, the serum and urine samples were diluted with phosphate buffer (pH 7) with a 1:50 ratio. The diluted samples were found to be FA free. Then known amounts of FA (5 and 10 µM) were spiked and analyzed. Amperometry experiments were carried out using GCE/rGO/PDA/AuNPs in FA-spiked biological samples. The added, found, and recovery values are presented in Table 4. The recovery values are in an acceptable range of 95.2%–97.3%, which indicates the potential practicality of the electrode.

## 3. Materials and Methods

### 3.1. Chemicals and Instrumentation

Multi-walled CNTs (bundled > 95%), graphite (powder, <20 μm), dopamine, HAuCl_4_·3H_2_O (99.9%), chloramphenicol, folic acid, and all other chemicals were acquired from sigma–Aldrich, Taiwan and used as received. All chemical reagents were used as received without further purification. Deionized water from Millipore was used for the reagent preparation, and throughout the experiments. Na_2_HPO_4_·12H_2_O and NaH_2_PO_4_·2H_2_O were used to prepare 0.1 M phosphate buffer (pH 7.0). Milk, powdered milk, and honey samples were purchased from a local supermarket, Taipei, Taiwan. Stock solution of CAP and FA were prepared in 0.1 M phosphate buffer (pH 7.0).

The size and morphology of the as-prepared materials were examined by using scanning electron microscopy (SEM, Hitachi S-3000 H scanning electron microscope) and transmission electron microscopy (TEM, Hitachi H-600 TEM). Samples for SEM and TEM were prepared by dropping an aqueous dispersion of materials on Indium tin oxide (ITO) substrates and copper TEM grids respectively, and dried under ambient conditions. Energy-dispersive X-ray (EDX) spectra and mapping were recorded using HORIBA EMAX X-ACT (Sensor + 24 V = 16 W, resolution at 5.9 keV). The powder X-ray diffraction (XRD) analysis was performed on a XPERT-PRO (PANalytical B.V., The Netherlands) diffractometer using Cu Kα radiation (*k* = 1.54 Å) to examine crystallinity of the nanomaterials. X-ray photoelectron spectra (XPS) were obtained by using XPS, PerkinElmer PHI–5702. 

### 3.2. Synthesis of CNTs/PDA/AuNPs

In a typical synthesis, first, functionalized CNTs were prepared by treating CNTs with acids. First, CNTs (50 mg) were suspended in a 100 mL mixture of H_2_SO_4_ and HNO_3_, ultrasonicated for 3 h, washed 2× with deionized water (DI) water (200 mL each) and 2× with ethanol (200 mL each), freeze-dried, and re-dispersed in water (1 mg mL^−1^). About 20 mL aqueous solution of 1 mg/mL DA prepared in Tris buffer was then added to 25 mL of the functionalized CNTs (*f*-CNTs). Subsequently, HAuCl_4_.3H_2_O (1 mL, 5 mM) was added and the entire mixture was stirred. Next, pH of the reaction mixture was adjusted to pH 9.0 by adding 0.1 M NaOH, and stirred for 15 min. Subsequently, the reaction mixture was transferred into a microwave oven, equipped with a temperature-control condenser system and a stirrer. Microwave with effective power set at 200 W for a total irradiation time was applied for 5 min and the solution was stirred at 500 rpm during irradiation. Finally, the CNTs/PDA/AuNPs nanocomposite was separated through centrifugation (4000 rpm, 30 min), washed 3× with DI water (50 mL each) and 2× with ethanol (50 mL each), and freeze-dried. 

### 3.3. Synthesis of rGO/PDA/AuNPs

First, graphite oxide was prepared from graphite by modified Hummers method. Then, it was exfoliated to GO in water via ultrasonic agitation for 2 h. About 50 mg of DA and HAuCl_4_·3H_2_O (1 mL, 5 mM) were added to the as-prepared GO (50 mL) and stirred for 15 min. Next, pH of the mixture was adjusted to pH 9.0 and stirred for 15 min. Subsequently, the reaction mixture was transformed into a microwave oven and irradiated for 5 min along with stirring (500 rpm). The nanocomposite was separated by centrifugation (4000 rpm, 30 min), washed 3× with DI water (50 mL each) and 2× with ethanol (50 mL each), and freeze-dried.

### 3.4. Fabrication of CNTs/PDA/AuNPs and rGO/PDA/AuNPs Modified Glassy Carbon Electrodes (GCEs)

1 mg of CNTs/PDA/AuNPs nanocomposite was re-dispersed in 1 mL water via ultrasonication for 10 min. 5 µL dispersion of CNTs/PDA/AuNPs was drop-casted on the pre-cleaned GCE and dried to yield GCE/CNTs/PDA/AuNPs. GCE/CNTs/AuNPs and GCE/CNTs/PDA were also prepared to execute control experiments. Similarly, GCE/rGO/PDA/AuNPs, GCE/rGO/AuNPs, and GCE/PDA/AuNPs were also fabricated.

### 3.5. Electrochemical Experiments

The electrochemical experiments were conducted in a conventional three-electrode system at room temperature with a potentiostat (CHI 621d workstation, USA). The nanocomposite-modified electrode, Ag/AgCl (saturated KCl), and Pt wire were used as working (area 0.071 cm^2^), reference, and counter electrodes, respectively. 0.1 M phosphate buffer (pH 7.0) was used as a supporting electrolyte for electrochemical studies. Before being modified with nanomaterials, the surface of the GCE was polished with 0.5 μm alumina slurry using a Buelher polishing pad, then ultrasonicated in ethanol and water for 5 min and dried under ambient conditions. The electrolyte solutions were deoxygenated with nitrogen for 10 min before each electrochemical test.

### 3.6. Preparation of Real Samples

The practical applicability of the method has been demonstrated in milk, powdered milk, and honey samples. The samples were prepared by following our previous procedures [28]. Powdered milk (1 g) was dissolved in 10 mL phosphate buffer (pH 7.0). Then, known amounts of CAP were spiked and the resulting solution was transferred to a microcentrifuge tube. Subsequently, 250 μL of perchloric acid (0.25 M) was added and the entire solution was stirred for 15 min and then centrifuged (3000× *g*, 10 min). The centrifugate was filtered, and pH was adjusted to 7 and used for real sample analysis of CAP. To prepare the honey sample, 1.0 g honey was dissolved in 10 mL phosphate buffer, transferred to a microcentrifuge tube, and spiked with CAP. About 4 mL of ethyl acetate was added and the solution was mixed completely on a vortex. The solution was centrifuged (3000× *g*, 10 min) and filtered. The filtrate was adjusted to pH 7 before being used for electrochemical analysis. To prepare the milk sample, 1 mL of milk in 5 mL phosphate buffer was spiked with appropriate amounts of CAP and used for studies. With his permission and approval from the organizational ethical clearance committee for human studies, human blood was obtained from a healthy volunteer. Human serum was extracted from blood by following the existing literature published elsewhere. 

## 4. Conclusions

In summary, an efficient, quick, green, and benign method for the synthesis of C/PDA/AuNPs nanocomposites has been developed. A series of morphological, elemental, spectral, and electrochemical characterizations ensures the successful formation of nanocomposites. Nano-sized spherical-shaped AuNPs in the size range of 50–100 nm, and thin rGO sheets at nanoscale were witnessed. The CNTs/PDA/AuNPs-modified electrode was found to be highly efficient for CAP sensing with a detection limit of 36 nM, and the method is successful in quantifying CAP spiked in milk, powdered milk. and honey samples (recoveries 95.0%–98.4%), thus it holds great promise in food safety appliances. The rGO/PDA/AuNPs nanocomposite is an excellent electrocatalyst for FA oxidation and it can be used to fabricate a rapid, sensitive sensor for FA in biological samples. The linear range for the FA sensor was 0.1–905 μM and the detection limit was 25 nM. The synthetic process described herein can be extended to produce multifunctional nanocomposites by replacing PDA with other polymers (water-soluble polymers) and AuNPs with other metal nanoparticles.

## Figures and Tables

**Table 1:** Comparison of analytical parameters for the determination of CAP at CNTs/PDA/AuNPs nanocomposite film with reported modifiers.

Electrode	Linear Range/µM	LOD/nM	Methods	Ref.
3D reduced GO	1–113	150	Differential pulse voltammetry	[42]
MoS_2_/self-doped polyaniline	0.1–1000	65	Differential pulse voltammetry	[43]
Fe_3_O_4_-carboxymethyl cellulose/Au	2.5–25	66	Square wave voltammetry	[44]
AuNPs/GO	1.5–2.95	250	Amperometry	[45]
Activated carbon fiber microelectrodes	0.1–10	47	Square wave voltammetry	[46]
Titanium nitride-rGO	0.05–100	20	Voltammetry	[47]
N-doped graphene/AuNPs	2–80	59	Linear sweep voltammetry	[30]
CNTs/PDA/AuNPs	0.1–534	36	Amperometry	This work

**Table 2:** Determination of spiked CAP in food samples using CNTs/PDA/AuNPs.

Samples	Added/µM	Found/µM	Recovery/%	RSD */%
Milk	1	0.96	96.0	2.88
	5	4.92	98.4	2.93
	10	9.63	96.3	3.50
Powdered milk	1	0.97	97.0	3.92
	5	4.83	96.6	3.52
	10	9.73	97.3	2.74
Honey	1	0.95	95.0	4.11
	5	4.80	96.0	3.4
	10	9.7	97.0	3.63

* Relative Standard Deviation (RSD) of three individual measurements.

**Table 3:** Comparison of folic acid analytical parameters between rGO/PDA/AuNPs film and reported modifiers.

Electrode	Linear Range/µM	LOD/µM	Ref.
Carbon paste electrode/Pt-Co nanoparticles/2-(3,4-dihydroxyphenethyl)isoindoline-1,3-dione	2–550	0.8	[48]
Methylene blue/rGO	4–167	0.5	[49]
Fe_3_O_4_ nanoparticles	0.065–98	0.002	[36]
B-doped polycrystalline diamond	0.1–167	0.03	[50]
MoS_2_/rGO	0.01–100	0.01	[51]
ferrocenedicarboxylic acid–MWCNTs	4.6–152	1.1	[52]
Au nanoclusters-activated graphene/MWCNT	10–170	0.09	[53]
rGO/PDA/AuNPs	0.1–905	0.025	This work

**Table 4:** Determination of spiked FA in biological samples using GCE/rGO/PDA/AuNPs.

Samples	Added/µM	Found/µM	Recovery/%	RSD */%
Human serum	5	4.76	95.2	2.58
	10	9.77	97.7	3.53
Human urine	5	4.85	97.0	2.63
	10	9.73	97.3	3.70

* Relative Standard Deviation (RSD) of three individual measurements.
